# A Comparative Analysis of NOX4 Protein Expression in Malignant and Non-Malignant Thyroid Tumors

**DOI:** 10.3390/cimb45070367

**Published:** 2023-07-13

**Authors:** Salma Fenniche, Mohamed Oukabli, Yassire Oubaddou, Hafsa Chahdi, Amal Damiri, Abir Alghuzlan, Abdelilah Laraqui, Nadia Dakka, Youssef Bakri, Corinne Dupuy, Rabii Ameziane El Hassani

**Affiliations:** 1 Laboratory of Biology of Human Pathologies (BioPatH), Faculty of Sciences, Mohammed V University in Rabat, Rabat 1014, Morocco; salma.fenniche@um5r.ac.ma (S.F.); yassire.oubaddou@um5r.ac.ma (Y.O.); n.dakka@um5r.ac.ma (N.D.); y.bakri@um5r.ac.ma (Y.B.); 2 Gustave Roussy Cancer Campus, Pavillon de Recherche N°2, F-94805 Villejuif, France; abir.alghuzlan@gustaveroussy.fr (A.A.); corinne.dupuy@gustaveroussy.fr (C.D.); 3 Faculty of Medicine and Pharmacy, University Paris-Saclay, F-91400 Orsay, France; 4 Unité Mixte de Recherche UMR9019 Centre National de la Recherche Scientifique, Pavillon de Recherche N°2, F-94805 Villejuif, France; 5 Service of Anatomical Pathology, Military Hospital of Instruction Mohammed V (HMIMV-R), Rabat 1014, Morocco; oukablimohamed@yahoo.fr (M.O.); hchahdi168@gmail.com (H.C.); amaldamiripath@gmail.com (A.D.); loranjad@yahoo.fr (A.L.); 6 Faculty of Medicine and Pharmacy, Mohammed V University in Rabat, Rabat 10001, Morocco

**Keywords:** papillary thyroid carcinomas, BRAF^V600E^ hot spot mutation, NADPH oxidase NOX4, biomarker

## Abstract

The comparative analysis of the expression of the reactive oxygen species-generating NADPH oxidase NOX4 from TCGA data shows that the NOX4 transcript is upregulated in papillary thyroid carcinomas (PTC)-BRAF^V600E^ tumors compared to PTC-BRAF^wt^ tumors. However, a comparative analysis of NOX4 at the protein level in malignant and non-malignant tumors is missing. We explored NOX4 protein expression by immunohistochemistry staining in malignant tumors (28 classical forms of PTC (C-PTC), 17 follicular variants of PTC (F-PTC), and three anaplastic thyroid carcinomas (ATCs)) and in non-malignant tumors (six lymphocytic thyroiditis, four Graves’ disease, ten goiters, and 20 hyperplasias). We detected the BRAF^V600E^ mutation by Sanger sequencing and digital droplet PCR. The results show that NOX4 was found to be higher (score ≥ 2) in C-PTC (92.9%) compared to F-PTC (52.9%) and ATC (33.3%) concerning malignant tumors. Interestingly, all C-PTC-BRAF^V600E^ expressed a high score for NOX4 at the protein level, strengthening the positive correlation between the BRAF^V600E^ mutation and NOX4 expression. In addition, independent of the mutational status of BRAF, we observed that 90% of C-PTC infiltrating tumors showed high NOX4 expression, suggesting that NOX4 may be considered a complementary biomarker in PTC aggressiveness. Interestingly, NOX4 was highly expressed in non-malignant thyroid diseases with different subcellular localizations.

## 1. Introduction

Thyroid cancer is the most common endocrine disease, and papillary thyroid carcinomas (PTC) are the most frequent forms of thyroid cancer from the follicular origin (78–90%), followed by follicular thyroid carcinomas (FTC) and anaplastic thyroid carcinomas (ATC) [1,2,3]. An alarming worldwide increase in PTC incidence has been reported, and the molecular/histological heterogeneity of PTC complicates their management from both diagnostic and prognostic perspectives [2,4,5,6,7,8,9]. The BRAF^V600E^ mutation is detected in 28–90% of PTC tumors, indicating that this driver mutation is a common event in these tumors [1,3,10,11,12,13,14,15,16].

BRAF^V600E^ is a potent activator of the MAPK pathway and is often associated with aggressiveness, metastasis, radioiodine refractoriness, and mortality in thyroid cancer [13,15,16,17,18]. BRAF^V600E^ is exclusively detected in PTC (mainly in classical PTC ‘C-PTC’ and rarely in the follicular variant of PTC ‘F-PTC’) and ATC, but never in FTC and rarely in benign thyroid adenomas [2,19,20]. BRAF^V600E^ is useful as a prognostic and predictive biomarker in managing thyroid cancer. Regarding 10-year survival, PTC and FTC have survival rates of about 90–98%, whereas poorly differentiated thyroid carcinomas (PDTC) and ATC have survival rates of 50% and <10%, respectively [1]. Interestingly, high-grade tumors (PDTC and ATC) can arise from PTC and FTC through progressive genetic alterations and dedifferentiation [1,21], suggesting that thyroid tumor dedifferentiation can occur independently of the BRAF^V600E^ mutation because FTC is always BRAF^wt^. In addition, the absence of a positive correlation between the BRAF^V600E^ mutation and thyroid tumor aggressiveness was reported [22,23,24,25]. An interesting classification of PTC has been proposed that groups PTC tumors independently of their molecular signature. PTC-BRAF^V600E^-like tumors, including PTC-BRAF^V600E^ and PTC-BRAF^wt^, share higher transcriptional MAPK pathway activity and higher dedifferentiation [7,26]. Consequently, exploring additional biomarkers other than BRAF^V600E^ could improve the management of PTC patients.

Reactive oxygen species (ROS) have been suspected of being involved in thyroid tumorigenesis for several years, and the role of ROS-generating NADPH oxidases has been analyzed in thyroid carcinomas [17,27,28]. Weyemi U et al. observed that NADPH oxidase 4 (NOX4) protein is overexpressed in thyroid cancer tissue (11 PTC) compared to normal adjacent tissue (eight NAT) [27]. Azouzi N et al. established a positive link between BRAF^V600E^ and NOX4 expression (at the mRNA level) by exploring about 500 PTC (BRAF^V600E^ versus BRAF^wt^) from the TCGA database ‘Genome Atlas’ [17]. This study explored NOX4 expression at the protein level in 134 thyroid tissues (48 malignant tissues, 46 normal tissues surrounding the tumors, and 40 non-malignant tissues). Our results showed that the NOX4 protein score is higher in PTC, and we strengthened the link between NOX4 protein expression and BRAF^V600E^ mutation in PTC. Interestingly, and unlike the mutational status of BRAF, NOX4 expression is higher in C-PTC infiltrating tumors, highlighting a potential role of NOX4 as a marker of aggressiveness in PTC. Finally, we observed an overexpression of NOX4 in non-malignant diseases with different subcellular localization.

## 2. Materials and Methods

This study was approved by the Ethics Committee for Biomedical Research (CERB) of the Faculty of Medicine and Pharmacy in Rabat, with approval number 52/20.

### 2.1. Study Subjects

In this retrospective study, we collected 134 FFPE thyroid tissues from patients diagnosed at the Department of Anatomical Pathology in Military Hospital of Instruction Mohammed V in Rabat (HMIMV-R) between January 2015 and December 2021. Our cohort included 46 NAT, 28 classical forms of papillary thyroid carcinomas (C-PTC), 17 follicular variants of papillary thyroid carcinomas (F-PTC), three ATC, and 40 non-malignant thyroid tissues (including six lymphocytic thyroiditis, four Graves’ disease, ten goiters, and 20 hyperplasias). Malignant and non-malignant thyroid tissues were histologically classified according to the World Health Organization (WHO) classification (Lloyd R.V. et al., 4th Edition, IARC: Lyon 2017) by an experienced anatomopathologist at the time of diagnosis. In addition, retrospective diagnosis confirmation was performed by experienced anatomopathologists from both HMIMV in Rabat (Morocco) and Gustave Roussy Cancer Institute (France). FFPE block selection was based on (1) cell sufficiency (more than 90% of tumor cells in thyroid tumors/absence of tumor cells in non-tumoral tissues) and (2) FFPE block availability.

The collected information from the medical files of the selected patients constituted a database for this study, providing all clinicopathological features of the patients.

### 2.2. NOX4 Immunohistochemistry Staining

Immunohistochemical staining of NOX4 was performed using rabbit polyclonal anti-NOX4 (ab154244, Abcam, Cambridge, United Kingdom), and Dako EnVision™ FLEX High pH ‘LINK’/FLEX IHC Microscope Slides (Agilent, Santa Clara, California, United States) in an automaton (Autostainer Link 48; Agilent) according to the manufacturer’s protocols. These antibodies (Abs), whose immunogen corresponds to human NOX4 (aa 252–564), are suitable for IHC staining of FFPE tissue (IHC-P). The validation of NOX4 staining was performed after testing several Ab dilutions (1:250, 1:500, 1:750 in EnVision™ FLEX Antibody Diluent) on normal human kidney tissue (FFPE Block) (Supplementary Figure S2). Counterstaining was performed by hematoxylin ‘EnVision™ FLEX Hematoxylin’. Immunostaining of thyroid tissue without NOX4 Ab served as a negative control.

An experienced anatomopathologist from HMIMV in Rabat and Gustave Roussy Cancer Institute (France) assigned two scores to NOX4 protein expression. A score <2 corresponds to an absence or low expression of NOX4 protein, and a score ≥2 corresponds to a medium or high expression of NOX4 protein. As usually used by trained pathologist, the score of the staining intensity could be negative (0), weak (1), moderate (2), and strong (3) [29]. For our study, we classified scores <2 (including 0 and 1) and ≥2 (including 2 and 3).

In addition, the subcellular localization of NOX4 was noted. Slides were scanned with a NanoZoomer HT C9600 (Hamamatsu Photonics KK, Hamamatsu, Japan) digital scanner, using a ×40 objective. Scanned slides were uploaded on the CaloPix database (Tribvn Healthcare, Chatillon, France).

### 2.3. Mutational Analysis

BRAF^V600E^ hotspot mutation was analyzed in 48 malignant thyroid tissues, including 28 C-PTCs, 17 F-PTCs, and three ATCs, using Sanger direct sequencing and digital droplet PCR after genomic DNA extraction.

The genomic DNA extraction was performed in 8 sections of 10 μm each, according to the manufacturer’s ‘QIAamp DNA FFPE Tissue Kit (Qiagen)’ protocol, specially designed for purifying DNA from FFPE tissue sections. The quality and concentration of the extracted DNA were determined using the IMPLEN NanoPhotometer N60.

After genomic DNA extraction, PCRs were conducted using the HotStart Taq polymerase (Qiagen) with the following conditions: 95 °C for 15 min, followed by 42 cycles of 95 °C for 30 s, 53 °C for 30 s, and 72 °C for 45 s, and a final extension cycle of 72 °C for 10 min, all carried out in the Thermal Cycler (ProFlex PCR System; Thermo Fisher, Waltham, MA, USA). The forward and reverse primers for BRAF Exon 15 were (F) 5′-TCA TAA TGC TTG CTC TGA TAG GA-3′ and (R) 5′-GGC CAA AAA TTT AAT CAG TGG A-3′. The positive PCR products for BRAF^V600E^, obtained after migration in 1.5% agarose gel electrophoresis, were purified using Exo-SAP before being bidirectionally sequenced with the Big Dye Terminator sequencing kit (Applied Biosystems, Foster City, CA, USA) at the UATRS Platform, CNRST, Rabat, Morocco. DNA sequences were analyzed with the SeqScape 2.7 software (Applied Biosystems, Waltham, MA, USA).

For digital droplet PCR, genomic DNA was extracted using the ‘Maxwell^®^ RSC DNA FFPE Kit’ from Promega/‘Maxwell^®^ RSC Instruments (Cat.# AS4500, AS8500)’ according to the manufacturer’s protocols (ASB1450). The extracted genomic DNA was sequenced using Droplet Digital PCR ‘ddPCR’ at the platform of molecular medicine in Gustave Roussy institute by determining the copy number of BRAF^V600E^ and BRAF^wt^ in each sample.

### 2.4. Statistical Analysis

All statistical analyses were performed using GraphPad Prism 8. Fisher’s exact and chi-square tests examined the associations between clinicopathological variables, BRAF^V600E^, NOX4 protein expression, and NOX4 protein localization. A *p*-value of <0.05 was considered statistically significant for all analyses.

## 3. Results

### 3.1. Clinicopathological Characteristics of Patients

The analysis of the clinicopathologic data of our cohort revealed a female predominance (81%) (Table 1). The median age at diagnosis was 42.5 years, with a standard error of 2.4 years. Histologically, PTC comprise several histological subtypes (variants) that share specific nuclear features of PTC. We observed that C-PTC constituted the most frequent histological subtype (58.33%), followed by F-PTC (35.41%) and finally ATCs (6.26%) (Table 1). Based on aggressive features, we found that most thyroid cancers presented with a tumor size greater than 1 cm, while we noted the presence of vascular emboli in 15% of cases, capsular breach in 23% of cases, and lymph node metastases in 6.3% of cases (Table 1).

### 3.2. Clinicopathological Parameters of Each Histological Subtype of Thyroid Carcinomas

The comparative analysis between the histological type of 48 thyroid carcinomas (45 PTCs and three ATCs) and their clinicopathological characteristics revealed that 46.4% of C-PTC occurs in individuals aged ≥45 years old. However, 52.9% of F-PTCs occur in individuals aged <45 years old, while 66.7% of ATCs occur in individuals aged >45 years old (see Table 2). Regarding tumor size, we observed that most PTC tumors were >1 cm (64.3% for C-PTCs and 70.6% for F-PTC). As for vascular emboli and capsular breach, we found them, respectively, in 33.3% and 0% of ATCs, 17.9% and 32.1% of C-PTC, and 5.9% and 11.8% of F-PTC (see Table 2). However, we detected lymph node metastasis only in the classical form of PTC (10.7%) (Table 2).

### 3.3. BRAF^V600E^ Mutation in Thyroid Carcinomas

**Figure 1:**
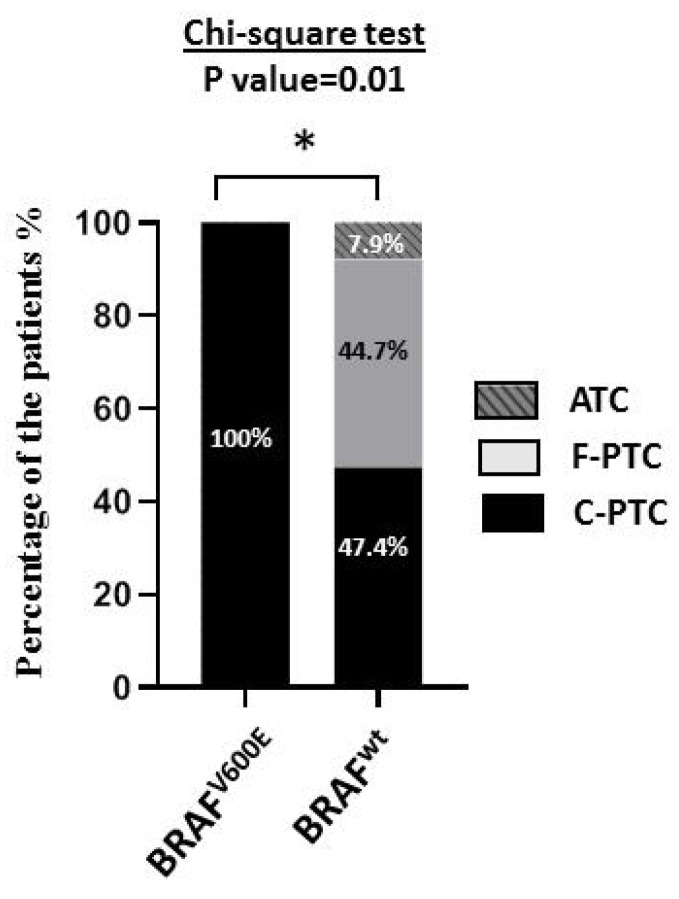
BRAF^V600E^ mutation in thyroid carcinomas. Association between BRAF^V600E^ mutation and histological type of thyroid carcinomas (*n* = 48). In total, 100% of thyroid carcinoma harboring BRAF^V600E^ are exclusively C-PTC. *ATC*: anaplastic thyroid carcinoma (*n* = 3). *C-PTC*: classical forms of papillary thyroid carcinoma (*n* = 28). *F-PTC*: follicular variants of papillary thyroid carcinoma (*n* = 17). BRAF^V600E^ (*n* = 10), BRAF^wt^ (*n* = 38). The statistical tests performed by GraphPad 8. * The statistical significance is affirmed by a *p*-value less than 0.05. *: *p*-value ≤ 0.05.

We analyzed the mutational profile of 48 thyroid carcinomas (45 PTC = 28 C-PTC + 17 F-PTC and three ATC) regarding the BRAF^V600E^ hotspot mutation using Sanger direct sequencing and digital droplet PCR (ddPCR). The mutational profiles of 41 samples (BRAF^V600E^ or BRAF^wt^) were detected by Sanger direct sequencing, while ddPCR explored 7 samples. Genomic DNA extracted from FFPE block tissues is known to be extensively fragmented [30,31,32]. Therefore, seven samples whose results were not exploitable by Sanger direct sequencing were analyzed by ddPCR. (Figure S1 Supplementary Materials) shows an example of electropherogram sequences (Figure S1a Supplementary Materials) and an example of results from ddPCR (Figure S1b Supplementary Materials) for BRAF^V600E^ mutation in C-PTC and BRAF^wt^ in F-PTC. In this study, BRAF^V600E^ mutation was found exclusively in the classical form of PTC (10 C-PTC-BRAF^V600E^/28 C-PTC: 35.7%), while no mutation was detected in the follicular variant of PTC and ATC (see Figure 1). This percentage is about 20.83% in all thyroid carcinomas (10 C-PTC-BRAF^V600E^/48; 48 = 28 C-PTC + 17 F-PTC + 3 ATC) and 22.22% in all papillary thyroid carcinomas (ten C-PTC-BRAF^V600E^/45; 45 = 28 C-PTC + 17 F-PTC). Finally, we did not observe any statistically significant association between BRAF^V600E^ mutation and the aggressiveness of thyroid carcinomas (see Table 3).

### 3.4. NOX4 Protein Expression in Human Malignant Thyroid Tissues

**Figure 2:**
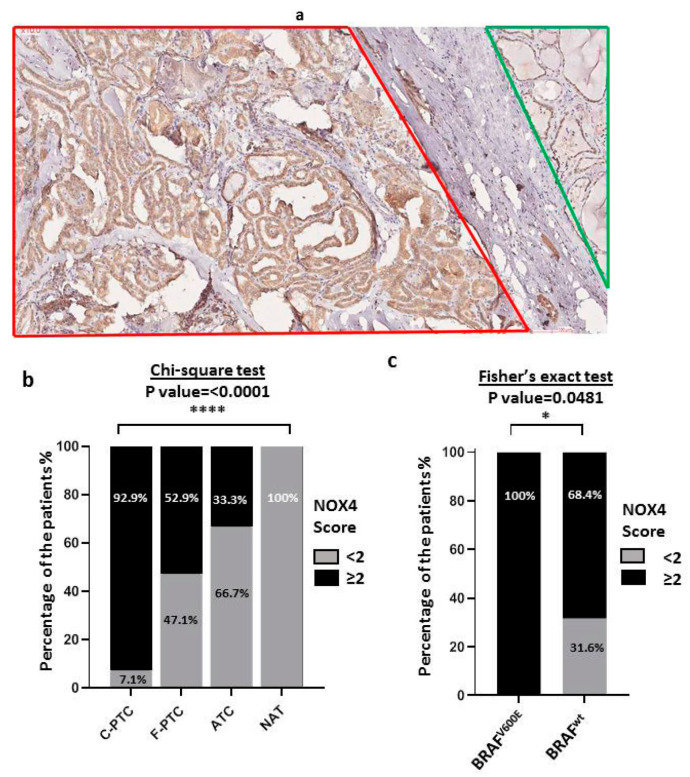
NOX4 protein expression in thyroid carcinomas. (**a**) Representative example of NOX4 protein expression in C-PTC. Red lines represent tumor tissue sections, and green lines represent normal adjacent tissue (NAT) sections on the same slide (×10). There is a high expression level of NOX4 protein in C-PTC and a low expression level of NOX4 in its adjacent normal tissue (NAT). (**b**) Comparative analysis of NOX4 protein expression in human thyroid tumors (*n* = 48:28 C-PTC, 17 F-PTC, 3 ATC) compared to their normal adjacent tissues (46 NAT). Percentage calculated according to the number of each score <2 and ≥2. The score <2 represents an absence or very low and low expression of NOX4 protein (*n* = 58). The score ≥2 represents a middle or/and high expression level of NOX4 protein (*n* = 36). (**c**) Association between BRAF^V600E^ mutation and NOX4 expression. In total, 100% of thyroid carcinoma harboring BRAF^V600E^ mutation showed high expression level of NOX4 protein. BRAF^V600E^ (*n* = 10), BRAF^wt^ (*n* = 38). The statistical tests are performed by GraphPad 8. * The statistical significance is affirmed by a *p*-value under 0.05. *: *p*-value ≤ 0.05, ****: *p*-value ≤ 0.0001.

The antibody specificity and dilution choice (1:250) were validated in human kidney tissues (Figure S2) known to express NOX4 protein [33]. NOX4 protein expression was analyzed by immunohistochemistry staining in thyroid tumor tissues and their paired NAT (Figure 2a). Our results showed that 92.8% of C-PTC, 52.9% of F-PTC, and 33.3% of ATC express high levels of NOX4 protein (score ≥ 2) (see Figure 2b). No high NOX4 protein expression was observed in normal adjacent tissues (see Figure 2b). Notably, most FFPE thyroid tumor blocks contain both tumor areas and their normal adjacent tissues NAT (42/46 NAT), enabling us to immunostain both tumor and normal adjacent tissues on the same slide (see Figure 2a). Importantly, all thyroid tumors (BRAF^V600E^) overexpressed NOX4 protein (100%: 10 C-PTC/10-C-PTC), highlighting the positive correlation between BRAF^V600E^ mutation and NOX4 protein expression in PTC (*p*-value < 0.0001) (see Figure 2c). However, a non-negligible percentage of BRAF^wt^ thyroid tumors (68.4%: 26/38) also exhibited a high level of NOX4 expression (see Figure 2c).

**Figure 3:**
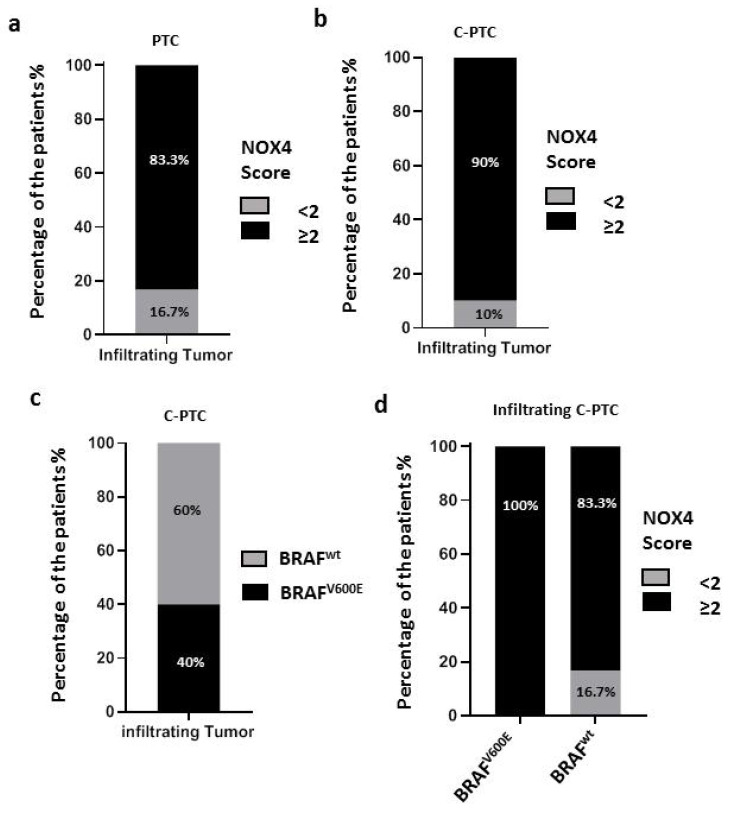
Correlation of infiltrating character followed by the limitation of the border of the tumor with NOX4 protein expression and BRAF^V600E^ mutation in PTC (*n* = 21: 17 c-PTC, 4 F-PTC). (**a**) In total, 83% (10/12) of papillary thyroid carcinoma with an infiltrating character show overexpression of NOX4 protein (*n* = 12). (**b**) C-PTC: high expression of NOX4 protein in infiltrating tumors (*n* = 10) (90% (9/10)) with a score ≥2. (**c**) C-PTC: 40% (4/10) of papillary thyroid carcinoma with an infiltrating character harbor BRAF^V600E^ mutation. (**d**) In total, 100% of infiltrating C-PTC harboring BRAF^V600E^ mutation overexpress NOX4 protein. Percentage calculated according to the total number of infiltrating tumors of each histological type.

To investigate the involvement of NOX4 protein in thyroid tumor aggressiveness, we observed a statistically significant correlation between the overexpression of NOX4 protein and the presence of a capsular breach, indicating the association of NOX4 protein with the aggressiveness of thyroid carcinomas in our cohort (*p*-value = 0.0481) (see Table 4). Additionally, we evaluated NOX4 protein expression in infiltrating PTC tumors, characterized by the infiltration of tumor borders and consequently presenting a risk of metastasis initiation. We found that the majority of PTC infiltrating tumors exhibited a high level of NOX4 protein expression (83.3%: score ≥2 for all PTC and 90% for C-PTC: score ≥ 2) (see Figure 3a,b), highlighting a potential role of NOX4 protein in PTC aggressiveness. However, only 40% of infiltrating tumors (C-PTC) were BRAF^V600E^ positive (see Figure 3c), while all these tumors overexpressed NOX4 (100% of C-PTC BRAF^V600E^: score ≥ 2) (see Figure 3d). Additionally, 83.3% of infiltrating C-PTC (BRAF^wt^) overexpressed NOX4 protein independently of the presence of the BRAF^V600E^ mutation (Figure 3d) (Fisher’s test: *p* ≥ 0.999 for BRAF^wt^ infiltrating C-PTC versus BRAF^V600E^ infiltrating C-PTC).

### 3.5. NOX4 Protein Expression and Subcellular Localization in Non-Malignant Human Thyroid Tissues

**Figure 4:**
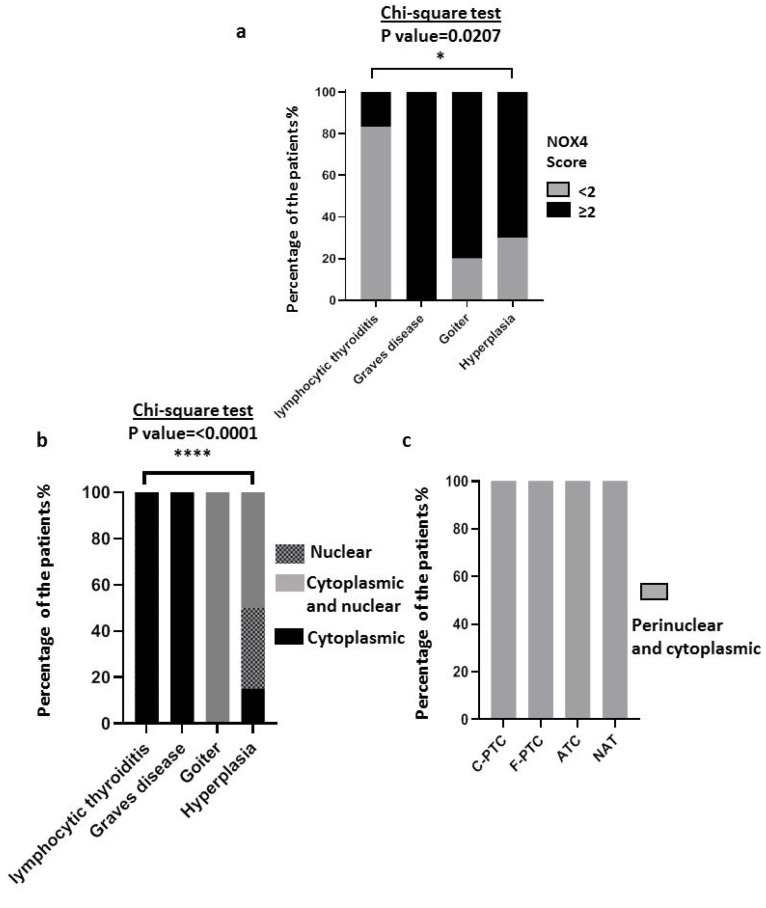
Immunohistochemical analysis of NOX4 protein expression and subcellular localization in thyroid carcinomas and non-malignant thyroid diseases. (**a**) NOX4 protein expression in 40 non-malignant thyroid diseases (six lymphocytic thyroiditis, four Graves’ disease, ten goiters, and 20 hyperplasias. (**b**) Subcellular localization of NOX4 protein in 40 non-malignant thyroid diseases (six lymphocytic thyroiditis, four Graves’ disease, ten goiters, and 20 hyperplasias). (**c**) Subcellular localization of NOX4 protein in 94 malignant thyroid tissues (28 c-PTC, 17 F-PTC, 3 ATC, and 46 NAT). The statistical tests performed by GraphPad 8. * The statistical significance is affirmed by a *p*-value less than 0.05. *: *p*-value ≤ 0.05, ****: *p*-value ≤ 0.0001.

We explored 40 non-malignant human thyroid disease tissues (six lymphocytic thyroiditis, four Graves’ disease, ten goiters, and 20 hyperplasias) and observed that the protein level of NOX4 was higher in Graves’ disease (100%: 4/4), goiters (80%: 8/10), and hyperplasias (70%: 14/20) (Figure 4a). Interestingly, we observed a different subcellular localization of NOX4 in non-malignant human thyroid tissues compared to malignant thyroid tissues (Figure 4c,d). NOX4 immunostaining revealed a perinuclear and cytoplasmic (intracellular) localization in both malignant thyroid tissues (100%: 48/48) and their normal adjacent tissues (100%: 46/46) (see Figure 4c). However, NOX4 localization was different in non-malignant thyroid tissues, being exclusively intracellular in both lymphocytic thyroiditis (100%: 6/6) and Graves’ disease (100%: 4/4), nuclear and intracellular in goiters (100%: 10/10), and at different localizations in hyperplasias: intracellular (15%: 3/20), nuclear (35%: 7/20), and intracellular and nuclear (50%: 10/20) (Figure 4b).

## 4. Discussion

PTC is a heterogeneous group of thyroid cancer, and its molecular and histological diversity constitute a real challenge for managing these tumors. Molecular exploration of new and/or complementary biomarkers could improve the management of PTC patients. ROS are involved in both physiological and pathological processes in the thyroid, and human cells can produce ROS through various enzymes, including NADPH oxidases (NOX1, NOX2, NOX3, NOX4, NOX5, DUOX1, and DUOX2). Thyrocytes express three NADPH oxidases (DUOX1, DUOX2, and NOX4) with different subcellular localizations and functions. In the thyroid gland, DUOX2 contributes mainly to thyroid hormone synthesis by providing H_2_O_2_ to thyroid peroxidase (TPO), while ROS-generating NADPH oxidases DUOX1 and NOX4 are associated with oxidative DNA damage that could promote thyroid radio-carcinogenesis and oncogenes thyroid cancer dedifferentiation, respectively [17,27,33,34,35].

Importantly, unlike the other NADPH oxidases, the level of NOX4 protein is directly related to the level of ROS generation. Therefore, a high level of NOX4 may promote oxidative DNA damage and genomic instability in thyroid cells [33]. In this study, we analyzed NOX4 expression at the protein level by immunostaining in 134 thyroid tissues (48 thyroid carcinomas, 46 normal adjacent tissues, and 40 non-malignant thyroid tissues) to improve our understanding of the potential role of NOX4 as a predictive marker of aggressiveness in PTC. Overall, we observed a high level of NOX4 protein in thyroid cancer tissues compared to normal adjacent tissues (92.9% of C-PTC, 52.9% of F-PTC, and 33.3% of ATC and 0% of NAT), which is consistent with previous reports with fewer tissues [28,36] (Figure 2b).

We previously showed a positive correlation between NOX4 mRNA and BRAF^V600E^ mutation in about 500 PTC from TCGA data [17], but this has not been established at the protein level in thyroid cancer tissues. In this study, all PTC-BRAF^V600E^ overexpressed NOX4 protein (Figure 2c). However, 68.4% of PTC-BRAF^wt^ also showed a high score of NOX4 protein (Figure 2c), emphasizing that NOX4 expression could be upregulated in thyroid cancer independently of BRAF mutational status. The TGF-β pathway plays a key role in thyroid tumorigenesis. The oncogenic effect of BRAF^V600E^ in thyroid cells was shown to be mediated in part by TGF-β [37], and NOX4 is upregulated in thyroid cancer cells under the control of the BRAF^V600E^-TGF-β axis [17]. The tumor microenvironment, including cancer-associated fibroblasts (CAF) and myeloid cells such as macrophages, also produces TGF-β [38]. Therefore, the microenvironment may contribute to inducing NOX4 in tumoral cells.

To establish the link between NOX4 protein and the infiltrating ability of PTC, we explored the presence or absence of a frank capsular breach, as its presence indicates the ability of tumors to extend/invade and promote extrathyroidal extension. More than 90% of C-PTC infiltrating tumors overexpressed NOX4 protein (score ≥ 2), suggesting a role of NOX4 in thyroid tumor aggressiveness (Figure 3).

Certainly, the expression level of NOX4 protein in terms of ROS production can be related to the incidence of oxidative DNA damage in thyrocytes. Therefore, the cellular localization of NOX4 appears to be of great interest. Indeed, the nuclear and perinuclear localization of ROS-generating NOX4 can promote genomic instability associated with thyrocyte transformation. The perinuclear localization of NOX4 observed in malignant human thyroid tissues (Figure 4) is concordant with this, as genomic instability is a characteristic of malignant tumors. Interestingly, goiter and hyperplasia tissues with a high score of NOX4 protein also showed nuclear and perinuclear localization (Figure 4), questioning the potential role of NOX4 in the progression of these non-malignant diseases to thyroid cancer. Indeed, a high risk of cancer was reported in goiters (18%) [39], and thyroid hyperplasia is reported as the most frequent benign disease associated with PTC [40]. Graves’ disease also shows a high level of NOX4 protein (Figure 4a), and this result can be explained by the constitutive activation of the TSH receptor (stimulating antibody, mutations) in Graves’ disease tissue [41]. The upregulation of NOX4 by TSH has previously been reported [27].

## 5. Conclusions

Taken together, the main conclusion of our study is that NOX4 expression could be a potential co-marker of thyroid cancer aggressiveness. However, complementary studies are needed to explore this possibility further. Indeed, BRAF^V600E^ mutation is not often associated with thyroid aggressiveness [22,23,24,25]. Several isoforms of NOX4 exist (from GenCards and Aceview), and an antibody recognizing the conserved catalytic core of NOX4 cannot discriminate between the different isoforms in thyroid tissues. Therefore, identifying NOX4 isoforms associated with different diseases may improve diagnosis and/or prognosis.

## Figures and Tables

**Table 1:** Clinicopathological parameters of patients with thyroid tumors (*n* = 48).

Clinicopathological Parameters	*n* (%)
**Gender**	
Female	39 (81%)
Male	9 (19%)
**Age**	
Median ± standard error	42.5 ± 2.4
**Histological variant**	
C-PTC	28/48 (58.33%)
F-PTC	17/48 (35.41%)
ATC	3/48 (6.26%)
**Tumor size**	
≤1 cm	7/48 (15%)
>1 cm	30/48 (63%)
unknown	11/48 (23%)
**vascular emboli**	
Presence	7/48 (15%)
Absence	41/48 (85%)
**Capsular breach**	
Presence	11/48 (23%)
Absence	37/48 (77%)
**lymph node metastasis**	
Presence	3/48 (6.3%)
Absence	34/48 (70.8%)
Unknown	11/48 (22.9%)

*PTC*: papillary thyroid carcinomas, *C-PTC*: classical form of papillary thyroid carcinomas, *F-PTC*: follicular variants of papillary thyroid carcinomas, *ATC*: anaplastic thyroid carcinomas. *Unknown*: absence of this information in both registers and medical files.

**Table 2:** Clinicopathological parameters of each histological subtype of thyroid carcinomas (*n* = 48).

Clinicopathological Parameters	PTC (*n* = 45)	C-PTC (*n* = 28)	F-PTC (*n* = 17)	ATC (*n* = 3)	C-PTC-F-PTC Chi-Square/Fisher’s Exact Test (*p*-Value < 0.05)
**Age**					0.1516
<45	19/45 (42.2%)	10/28 (35.7%)	9/17 (52.9%)	1/3 (33.3%)	
≥45	16/45 (35.6%)	13/28 (46.4%)	3/17 (17.7%)	2/3 (66.7%)	
unknown	10/45 (22.2%)	5/28 (17.9%)	5/17 (29.4%)	-	
**Tumor size**					>0.9999
≤1 cm	7/45 (15.6%)	4/28 (14.3%)	3/17 (17.6%)	-	
>1 cm	30/45 (66.7%)	18/28 (64.3%)	12/17 (70.6%)	-	
Unknown	8/45 (17.7%)	6/28 (21.4%)	2/17 (11.8%)	3 (100%)	
**vascular emboli**					0.3846
Presence	6/45 (13.3%)	5/28 (17.9%)	1/17 (5.9%)	1/3 (33.3%)	
Absence	39/45 (86.7%)	23/28 (82.1%)	16/17 (94.1%)	2/3 (66.7%)	
**Capsular breach**					0.1647
Presence	11/45 (24.4%)	9/28 (32.1%)	2/17 (11.8%)	0 (0%)	
Absence	34/45 (75.6%)	19/28 (67.9%)	15/17 (88.2%)	3/3 (100%)	
**lymph node metastasis**					0.2432
Presence	3/45 (6.6%)	3/28 (10.7%)	0 (0%)	0 (0%)	
Absence	34/45 (75.6%)	18/28 (64.2%)	16/17 (94.1%)	0 (0%)	
Unknown	8/45 (17.8%)	7/28 (25%)	1/17 (5.9%)	3/3 (100%)	

*PTC*: papillary thyroid carcinomas, *C-PTC*: classical form of papillary thyroid carcinomas, *F-PTC*: follicular variants of papillary thyroid carcinomas, *ATC*: anaplastic thyroid carcinomas. *Unknown*: absence of this information in both registers and medical files.

**Table 3:** BRAF^V600E^ mutation and C-PTC clinicopathological parameters (*n* = 28).

Clinicopathological Parameters	C-PTC BRAF^V600E^ (*n* = 10)	C-PTC BRAF^wt^ (*n* = 18)	Total	Chi-Square/Fisher’s Exact Test (*p*-Value < 0.05)
**Tumor size**				>0.9999
≤1 cm	1/10 (10%)	2/18 (11.1%)	3	
>1 cm	7/10 (70%)	12/18 (66.7%)	19	
unknown	2/10 (20%)	4/18 (22.2%)	6	
**lymph node metastasis**				>0.9999
Presence	1/10 (10%)	2/18 (11.1%)	3	
Absence	8/10 (80%)	10/18 (55.6%)	18	
unknown	1/10 (10%)	6/18 (33.3%)	7	
**vascular emboli**				0.8253
Presence	2/10 (20%)	3/18 (16.7%)	5	
Absence	8/10 (80%)	15/18 (83.3%)	23	
**Capsular breach**				0.5070
Presence	4/10 (40%)	5/18 (27.8%)	9	
Absence	6/10 (60%)	13/18 (72.2%)	19	

*C-PTC*: Classical form of papillary thyroid carcinomas. *Unknown*: absence of this information in both registers and medical files.

**Table 4:** NOX4 protein expression and clinicopathological parameters of thyroid tumors.

Clinicopathological Parameters	*n* (%)	NOX4 Overexpression (Score: ≥2)	NOX4 Low Expression (Score: <2)	Fisher’s Exact Test (*p*-Value < 0.05)
**Gender**				>0.9999
Female	39/48 (81%)	30/39 (76.9%)	9/39 (23.1%)	
Male	9/48 (19%)	7/9 (77.8%)	2/9 (22.2%)	
**Histological variant**				<0.0001
C-PTC	28/48 (58.33%)	26/28 (92.9%)	2/28 (7.1%)	
F-PTC	17/48 (35.41%)	9/17 (52.9%)	8/17 (47.1%)	
ATC	3/48 (6.26%)	1/3 (33.3%)	2/3 (66.7%)	
**Tumor size**				0.3079
≤1 cm	7/48 (15%)	7/7 (100%)	0 (0%)	
>1 cm	30/48 (63%)	22/30 (73.3%)	8/30 (26.7%)	
unknown	11/48 (23%)	7/11 (63.4%)	4/11 (36.6%)	
**vascular emboli**				
Presence	7/48 (15%)	6/7 (85.7%)	1/7 (14.3%)	
Absence	41/48 (85%)	30/41 (73.2%)	11/41 (26.8%)	0.6621
**Capsular breach**				
Presence	11/48 (23%)	11/11 (100%)	0/11 (0%)	
Absence	37/48 (77%)	25/37 (67.6%)	12/37 (32.4%)	0.0442
**lymph node metastasis**				0.5483
Presence	3/48 (6.3%)	3/3 (100%)	0/3 (0%)	
Absence	34/48 (70.8%)	24/34 (70.6%)	10/34 (29.4%)	
unknown	11/48 (22.9%)	9/11 (81.8%)	2/11 (18.2%)	

*PTC*: papillary thyroid carcinomas, *C-PTC*: classical form of papillary thyroid carcinomas, *F-PTC*: follicular variant of papillary thyroid carcinomas, *ATC*: anaplastic thyroid carcinomas. *NOX4*: NADPH oxidase 4. *Unknown*: absence of this information in both registers and medical files.

## Data Availability

Archived datasets are not available due to the ethic restriction.

## Supplementary Materials

The following supporting information can be downloaded at: https://www.mdpi.com/article/10.3390/cimb45070367/s1, Figure S1: An example of electropherogram sequences (Figure S1a) and an example of results from ddPCR (Figure S1b) for BRAF^V600E^ mutation in C-PTC and BRAF^wt^ in F-PTC. Figure S2: NOX4 Immunoexpression in normal kidney cells. Validation of anti-NOX4 antibody (ab154244) using different dilutions test (1:250,1:500 and 1:750).
