# Supplementary material for: A Comparative Analysis of NOX4 Protein Expression in Malignant and Non-Malignant Thyroid Tumors
Source: Curr Issues Mol Biol. 2023 Jul 13;45(7):5811–23. doi: 10.3390/cimb45070367 (PMC10378117; doi:10.3390/cimb45070367)
Supplement: Supplementary file 1 [file cimb-45-00367-s001.zip › cimb-2430226-supplementary.pptx]

## Slide 1
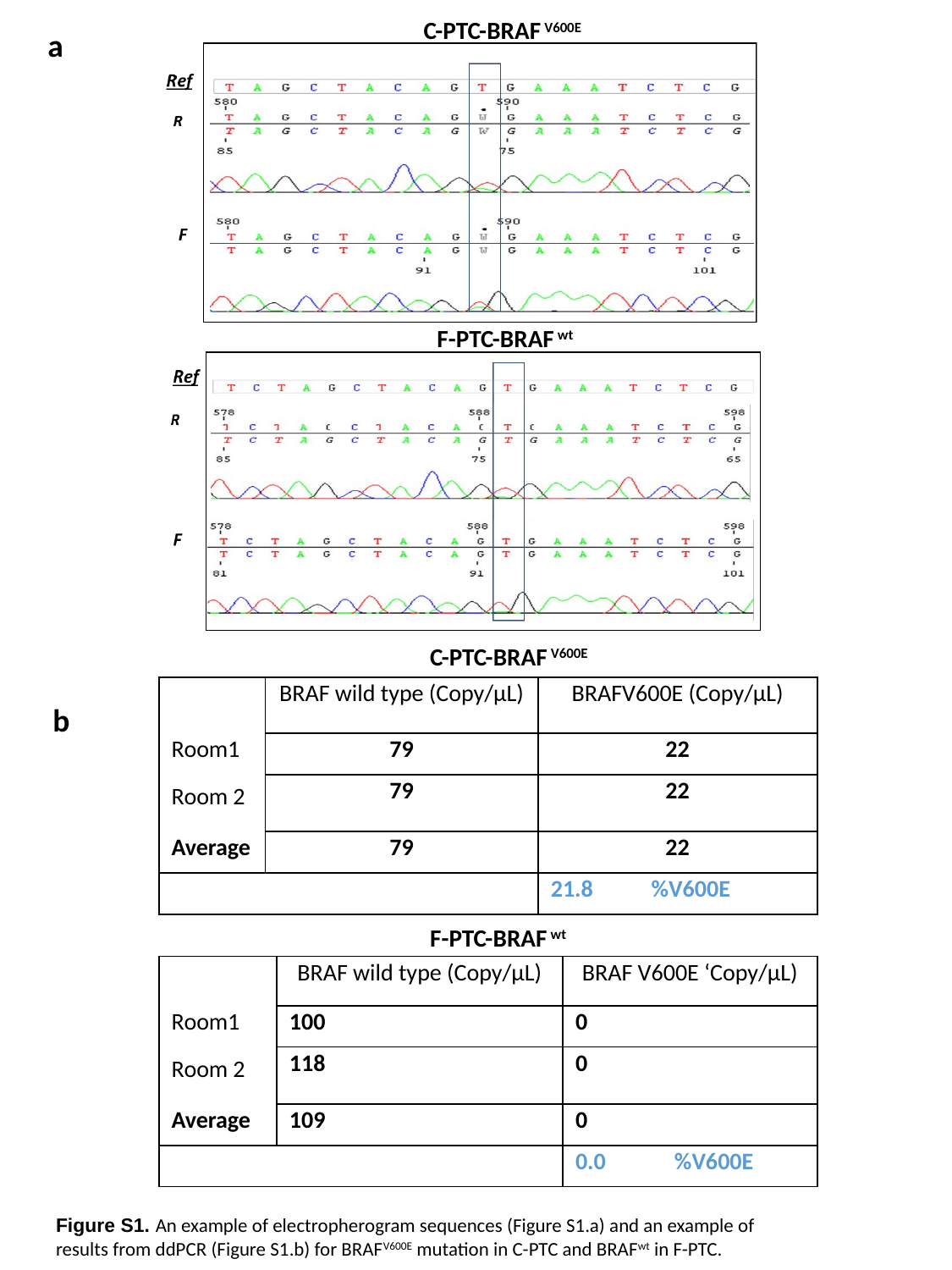

C-PTC-BRAF V600E
a
F-PTC-BRAF wt
C-PTC-BRAF V600E
| | BRAF wild type (Copy/µL) | BRAFV600E (Copy/µL) |
| --- | --- | --- |
| Room1 Room 2 | 79 | 22 |
| | 79 | 22 |
| Average | 79 | 22 |
| | | 21.8 %V600E |
b
F-PTC-BRAF wt
| | BRAF wild type (Copy/µL) | BRAF V600E ‘Copy/µL) |
| --- | --- | --- |
| Room1 Room 2 | 100 | 0 |
| | 118 | 0 |
| Average | 109 | 0 |
| | | 0.0 %V600E |
Figure S1. An example of electropherogram sequences (Figure S1.a) and an example of
results from ddPCR (Figure S1.b) for BRAFV600E mutation in C-PTC and BRAFwt in F-PTC.

## Slide 2
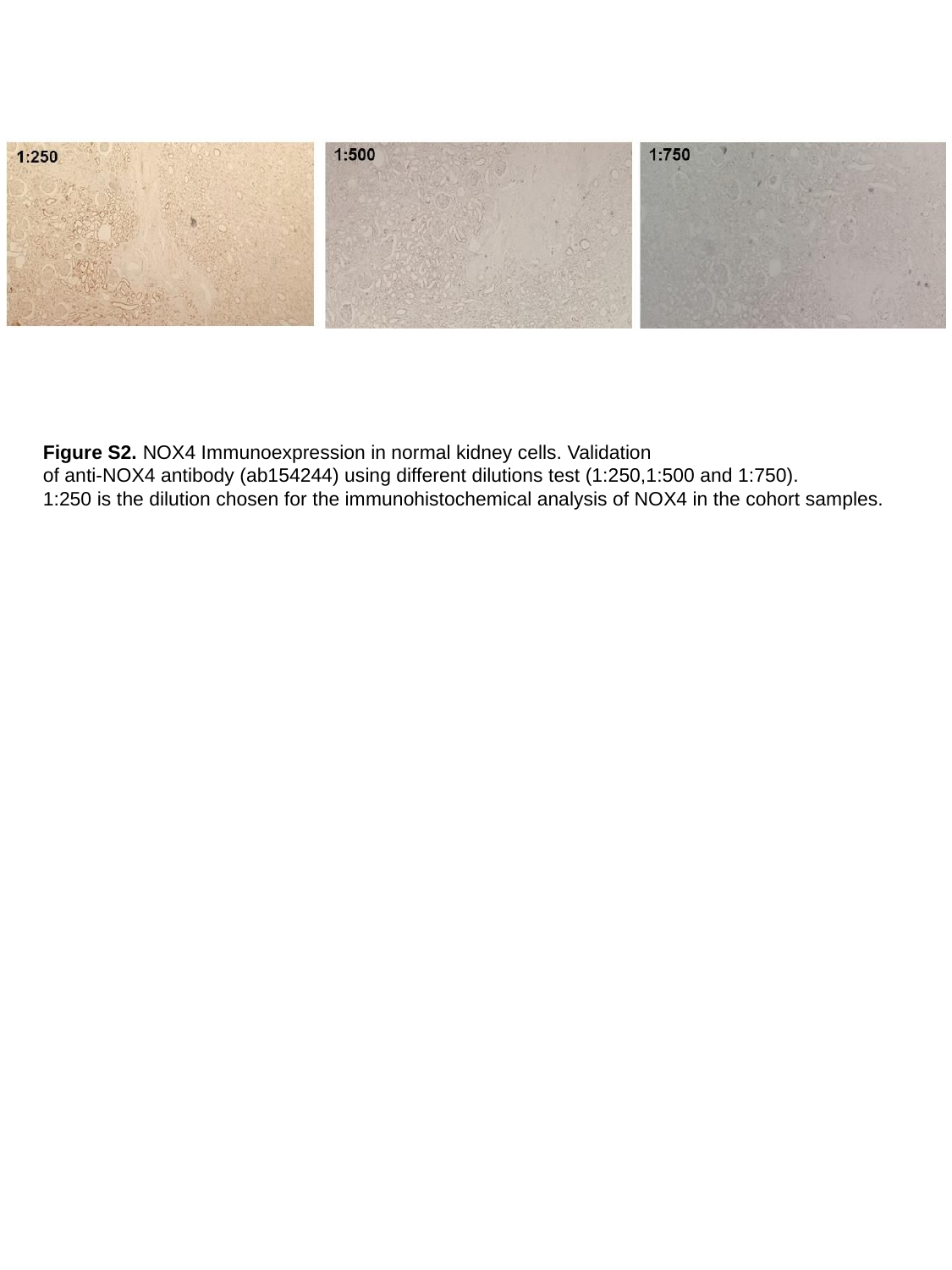

Figure S2. NOX4 Immunoexpression in normal kidney cells. Validation
of anti-NOX4 antibody (ab154244) using different dilutions test (1:250,1:500 and 1:750).
1:250 is the dilution chosen for the immunohistochemical analysis of NOX4 in the cohort samples.
